# Comparison of the Antennal Sensilla Ultrastructure of Two Cryptic Species in *Bemisia tabaci*

**DOI:** 10.1371/journal.pone.0121820

**Published:** 2015-03-30

**Authors:** Xiao-Man Zhang, Su Wang, Shu Li, Chen Luo, Yuan-Xi Li, Fan Zhang

**Affiliations:** 1 Institute of Plant and Environment Protection, Beijing Academy of Agriculture and Forestry Sciences, Beijing, China; 2 Key Laboratory of Integrated Management of Crop Diseases and Pest, Ministry of Education, Nanjing Agricultural University, Nanjing, Jiangsu, China; Institute of Zoology, CHINA

## Abstract

*Bemisia tabaci* is an important agricultural pest with worldwide distribution and host preference. Therefore, understanding the biology of this pest is important to devise specific pest control strategies. The antennae of herbivorous insects play an important role in the identification of hosts using plant volatiles. To understand the features of antennae in *B*. *tabaci* MEAM 1(formerly known as biotype ‘B’) and MED (formerly known as biotype ‘Q’), the morphology and distribution of the antennal sensilla were examined using scanning electron micrographs. The results showed that the average antennae length in MEAM 1 was longer than MED. No differences were observed in the number and distribution of antennal sensilla in MEAM 1 and MED antennae; each antenna had nine different types of sensilla. Both cryptic species possessed Microtrichia, Grooved surface trichodea sensilla, Chaetae sensilla, Coeloconic sensillaⅠandⅡ, Basiconic sensilla Ⅰ, Ⅱ and Ⅲ and Finger-like sensilla. This is the first report of Grooved surface trichodea sensilla and Basiconic sensilla Ⅱ on *B*. *tabaci* flies. The numbers of Chaetae sensilla were different in the females and males of MEAM 1 and MED, which females having 5 and males containing 7. The surface structure of Basiconic sensilla Ⅰ was different with MEAM 1 showing a multiple-pitted linen surface and MED showing a multiple-pitted pocking surface. Basiconic sensillaⅡ were double in one socket with the longer one having a multiple-pitted surface and the shorter one with a smooth surface. Basiconic Ⅲ and Finger-like sensillae were longer in MEAM 1 antennae than in MED antennae. Our results are expected to further the studies that link morphological characteristics to insect behavior and help devise strategies to control insect pests.

## Introduction

The tobacco whitefly, *Bemisia tabaci* (Gennadius), is a cryptic species complex that contains more than 28 morphologically indistinguishable species [1, 2]. Within this whitefly complex the Middle East-Asia Minor 1 (MEAM 1, formerly known as biotype ‘B’) and Mediterranean (MED, formerly known as biotype ‘Q’) are two destructive pests that destroy vegetables, fields and ornamental crops because they are vectors of geminiviruses, and have a broad host range, rapid dispersal, and ability to rapidly develop resistance to insecticides [1, 3–6]. Because of the broad host range and aggravated damage to a multitude of crops, the biology of *B*. *tabaci* in different host plants has been well studied [7, 8]. To reduce whitefly population, whitefly tropism and repellent plants have been used [2, 9]. However, the mechanism by which *B*. *tabaci* orients and identifies its host is not well understood. This line of study is critical to regulate the behavior of *B*. *tabaci* and to devise novel methods for integrated pest management.

Studies on the interaction between insects and host plant volatiles have shown the critical role of an insects’ olfactory system in finding host plants, mating, and spawning [10–12]. Antennae are the main olfactory organs in insects with an olfactory receptor (sensilla) system, which houses the neuronal receptors for volatiles. Therefore, analyzing the morphology and structure of sensilla is important to explore olfactory behavior and host identification mechanisms in insects.

Previous studies have described the external morphology and ultrastructure of *B*. *tabaci* sensilla [13–15]. However, these studies were carried out without discriminating cryptic species. Although the antennal sensilla ultrastructure of one of the *B*. *tabaci* cryptic species, MEAM 1, has been reported [16] the morphology and antennae structure in this species and others are not described.

In this study, we describe the fine external structure of the antennae and distribution of the antennal sensilla in the male and female adults of *B*. *tabaci* MEAM1 and MED cryptic species using scanning electron microscopy. The results presented here could further the study of olfactory mechanism in insects and provide the basis to link morphology to insect behavior and to study taxonomy in *B*. *tabaci* cryptic species.

## Materials & Methods

Colonies of two cryptic *B*. *tabaci* species, MEAM1 and MED, were obtained from the Institute of Vegetables and Flowers in the Chinese Academy of Agricultural Sciences, and established in the laboratory at the Institute of Plant and Environment Protection, Beijing Academy of Agriculture and Forestry Sciences, China. All colonies were maintained on cotton plants (*Gossypium hirsutum* L. var. ‘Shiyuan 321’) under a 16 h: 8 h, light: dark photoperiod at 25–28°C and 60–80% humidity. Adult whiteflies were anaesthetized at 4°C to separate males and females through a microscope (Nikon SMZ 1500), and transferred to 1.5 mL centrifuge tubes for further analyses. Adult whiteflies used for the scanning electron microscopy were less than 7 days old.

In each cryptic species, 30 individuals of each sex were transferred individually into 1.5 mL centrifuge tubes and rinsed three times in phosphate-buffered saline (PBS) pH 7.0 for 15 min each, and placed in 2.5% glutaraldehyde at 4°C overnight. Then, the samples were washed three times in 0.1 M pH 7.0 phosphate buffer for 15 min each, dehydrated in a graded ethanol series by incubating in 30, 50, 70, 80, 90, and 95% ethanol for 10 min each, and a final incubation in absolute ethanol for 15 min. The dehydrated specimen were soaked in isoamyl acetate for 15 min, and dried in Critical Point Dryer(LEICA-EM-SCD050)for 1.5 h. The antennae were carefully dissected from these individuals, mounted on stubs, and examined using an MZ205A stereomicroscope (Leica, Wetzlar, Germany). Then, the antennae were coated with 100 nm gold using a Leica sputtering ion exchanger (LEICA-EM-CPD300), and examined by scanning electron microscopy (SEM, FEI-Quanta-450 FEG, quanta, Germany).

Numbers and sizes of the various sensilla in the antennal segments were measured using Photoshop CS3 (Adobe System, Mountain View, CA, USA) based on the SEM photomicrographs of the antennal dorsal and ventral surfaces.

Statistical analysis: Sizes of the various sensilla in the antennal segments of the two *B*. *tabaci* cryptic species were analyzed using one-way analysis of variance (ANOVA). Means were separated using the least significant difference (LSD) test after a significant *F*-test at *P* > 0.05 (SAS Institute, 2008).

## Results

### Gross antennal morphology in MEAM1 and MED

**Fig 1:**
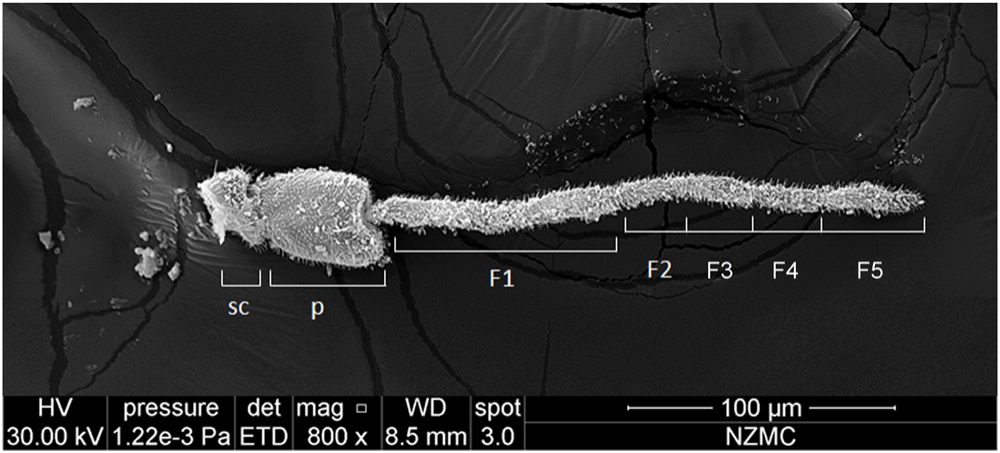
Antenna in female *Bemisia tabaci*, showing scape (SC), pedicel (P), and flagellum with 5 sub-segments. The antennae from the two *B*. *tabaci* cryptic species were the same, only MEAM1 female antenna is shown here.

**Fig 2:**
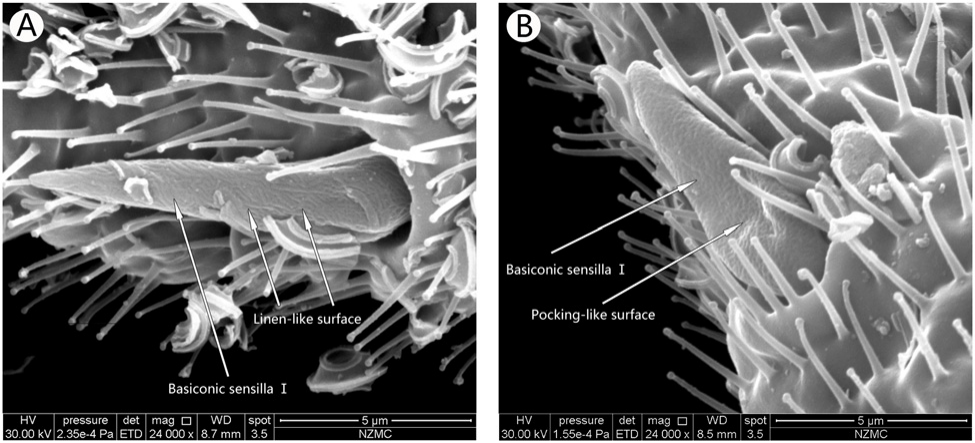
Ventral surface of the first flagellum showing the basiconic sensilla Ⅰ in *B*. *tabaci* MEAM1 male (A) and MED female (B). Arrows indicate the surface Basiconic sensilla I. MEAM1 (A) has linen-like surface, while MED (B) has multiple pocking-like surface.

**Fig 3:**
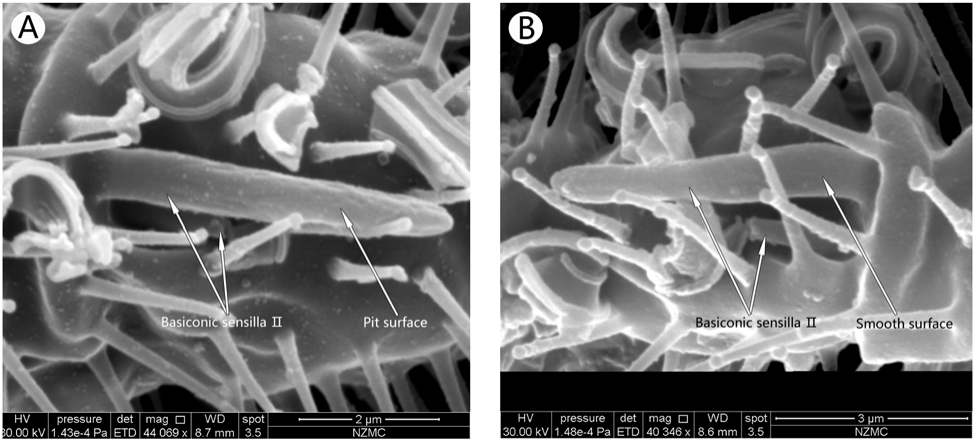
Ventral surface of the fourth flagellum, showing the basiconic sensilla Ⅱ in MEAM1 male (A) and MED female (B). Both have double Basiconic sensilla, one with smooth surface and the other has pit surface.

**Fig 4:**
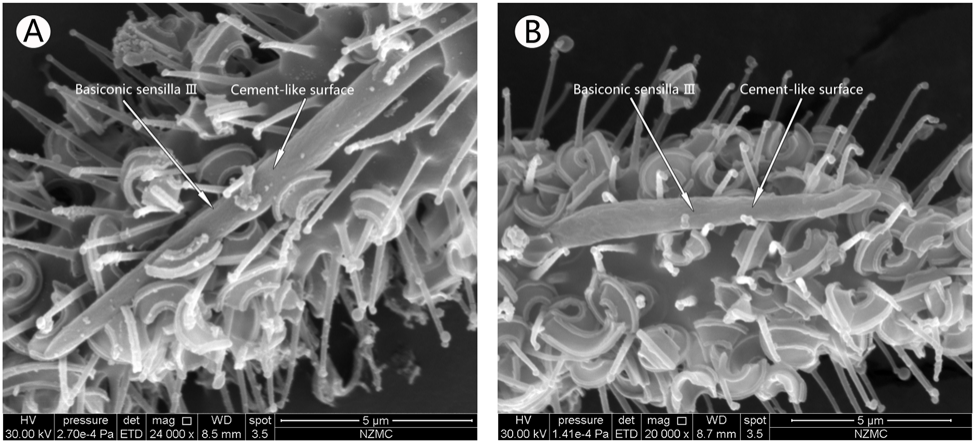
Ventral surface of the fifth flagellum, showing Basiconic sensilla Ⅲ in MEAM1 female (A) and MED female (B). Arrows indicate Basiconic sensilla with cement-like surface.

**Fig 5:**
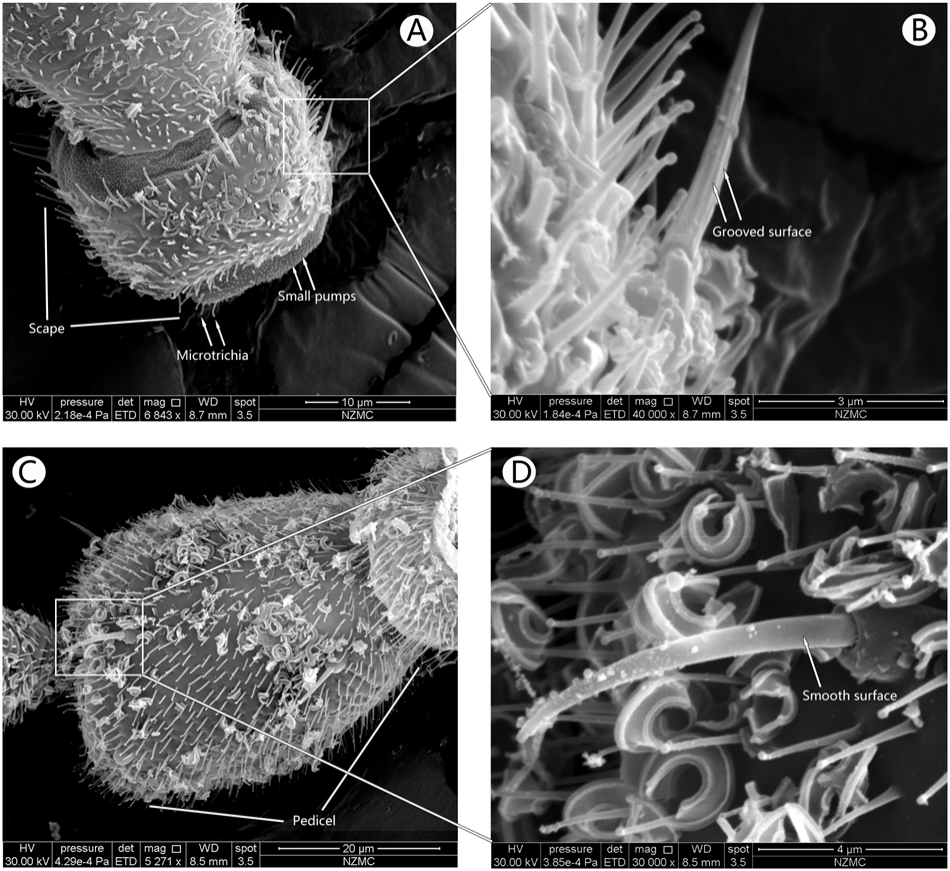
Ventral surface of the scape and pedicel. Scape (A) and Grooved surface trichodea sensilla (B) in MEAM1 male, which has grooved surface. Ventral surface of the bulbous pedicel, showing the Chaetae sensilla with smooth surface in MEAM1 female (C, D).

**Fig 6:**
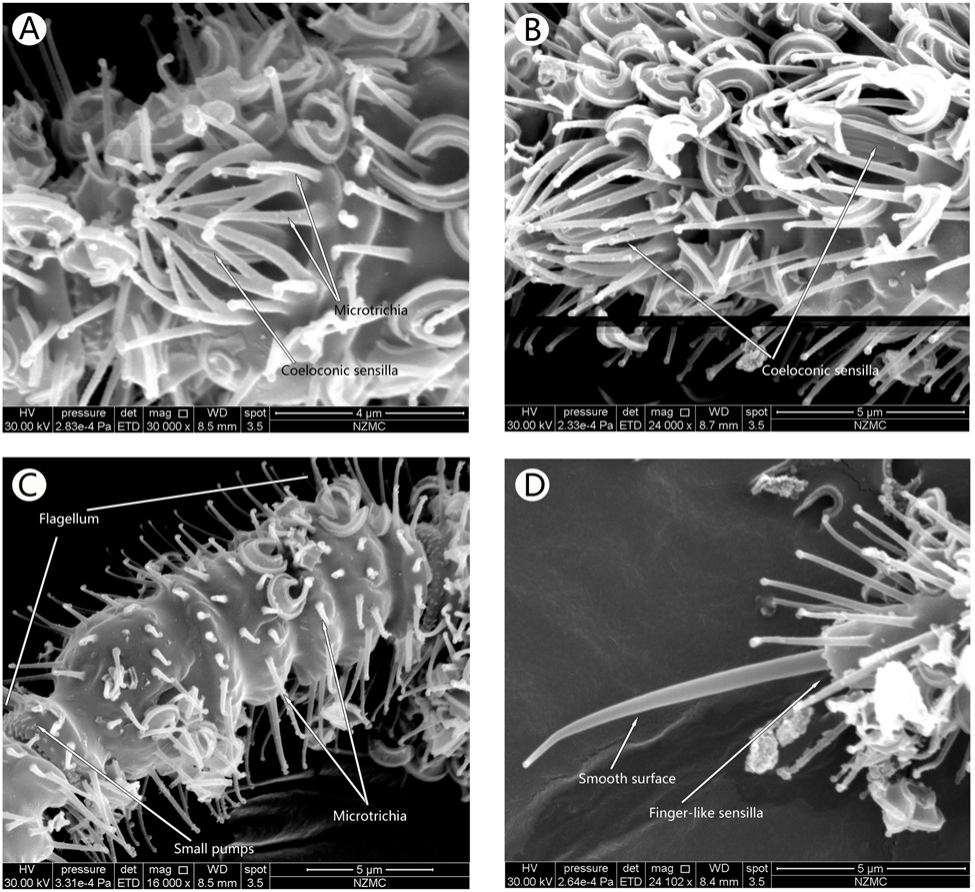
Coeloconic sensillaⅠandⅡ, Microtrichia sensilla and Finger-like sensilla of MEAM1. Ventral surface of the third flagellum showing the Coeloconic sensilla I in MEAM1 female (A). Ventral surface of the first flagellum with the Coeloconic sensilla II in MEAM1 male (B). Microtrichia sensilla in the second flagellum of MEAM1 male (C) and Finger-like sensilla in the fifth flagellum of MEAM1 male (D).

SEM analysis showed that the antennae in the adults of both cryptic species, MEAM1 and MED, had three segments, including a basal scape, a bulbous pedicel, and a long flagellum, Flagellar segments 1–5 (F1–F5) (Fig 1). Each flagellum was composed of 5 sub-segments. The types of sensilla identified were Microtrichia sensilla (MT), Basiconic sensilla (BA) (Figs 2–4), Grooved surface trichodea sensilla (GT) (Fig 5A and 5B), Chaetae sensilla (CH) (Fig 5C and 5D), Coeloconic sensilla (CO) (Fig 6A and 6B), and Finger-like sensilla (FS) (Fig 6C and 6D). The mean antennal length in MEAM1 females and males was 294.02±5.19 μm and 274.00±8.17 μm, respectively and in MED females and males it was 283.08±3.78 μm and 247.37±2.67 μm, respectively. Overall, the antennae in females were significantly longer than in males (Table 1). In addition, mean length of the segment scape was about 15 μm in both females and males (Table 1). The mean pedicel length in male MED was 40.98±1.18 μm, which was significantly shorter than the females. Similarly, the first sub-segment in the flagellum of MED male was 86.01±1.27 μm, which was shorter than the females. The second sub-segment in the flagellum was around 18 μm long, and the third was around 30 μm long in the females and males of both cryptic species. The fourth sub-segment in the flagellum of females (MEAM 1: 26.53±0.50 μm, MED: 26.95±0.84 μm) was significantly longer than in males (MEAM 1: 24.04±0.97 μm, MED: 23.82±0.28 μm). Similarly, the fifth sub-segment in the flagellum in *B*. *tabaci* MEAM 1 females (46.28±0.83 μm) was significantly longer than MEAM1 males (40.50±1.62 μm) and MED females (41.49±1.95 μm), which was significantly longer than MED males (34.88±1.88 μm).

**Table 1:** Lengths of the scape, pedicel, and flagellum in the two cryptic *B*. *tabaci* specie (mean±SD μm).

Segment	MEAM 1 female	MEAM 1 male	MED female	MED male
Scape	15.31±0.44^a^	14.82±0.361^a^	15.35±0.83^a^	15.09±0.81^a^
Pedicel	44.82±1.18^a^	42.25±1.18^a^	44.72±0.24^a^	40.98±1.18^b^
F_1_	99.88±4.01^a^	95.49±0.63^a^	97.32±2.20^a^	86.01±1.27^b^
F_2_	18.47±0.72^a^	17.13±0.98^a^	18.40±1.04^a^	18.57±0.96^a^
F_3_	31.30±1.54^a^	28.53±0.34^a^	31.85±0.26^a^	28.69±1.66^a^
F_4_	26.53±0.50^a^	24.04±0.97^b^	26.95±0.84^a^	23.82±0.28^b^
F_5_	46.28±0.83^a^	40.50±1.62^b^	41.49±1.95^b^	34.88±1.88^c^
Total	294.02±5.19^a^	274.00±8.17^b^	283.08±3.78^a^	247.37±2.67^c^

The same lowercase letters followed by mean lengths or widths indicate no significant difference at P>0.05

The length of the intersegmental gap from the segment-tip to the segment was 0.65 μm. The surface of the intersegmental gap had many small bumps, and extended to the inside of the segment (Fig 6A and 6C). The junction between the pedicel and the first flagella was unevenly thick, and the junctions between the sub-segments were evenly thick. The fifth sub-segment, however, had a tapering segment-tip (Fig 1).

On the surface of the junction between the head and the scape several small bumps were observed with MT among the bumps. MT were not present in other junctions, but were found only between the head and the scape (Fig 6A and 6C). The end of one sub-segment inserted into the tip of the following sub-segment thus interlocking the sub-segments.

### Characterization of sensilla

#### Types of sensilla

Six different types of sensilla were observed on the antennae of the two cryptic whitefly species females and males. They were MT, GT, CH, CO I and II, BA I, II and III, and FL. All sensilla were mostly distributed on the ventral surface of the antennae. Both females and males of the two cryptic species had the same types of sensilla. The numbers and distribution of 5 sensilla except the CH, in females and males of MEAM1 and MED were similar (Table 2). With respect to the CH, 5 were observed in females, and 7 in males.

**Table 2:** The prevalence of antennal sensilla in female and male MEAM1 and MED.

Type of sensilla	MT	GT	CH	CO	BA	FS
MEAM1 female	more	1	5	4	3	1
male	more	1	7	4	3	1
MED female	more	1	5	4	3	1
male	more	1	7	4	3	1

Abbreviation: MT: Microtrichia sensilla, GT: Grooved surface trichodea sensilla, CH: Chaetae sensilla, CO: Coeloconic sensilla, BA: Basiconic sensilla, FS: Finger-like sensilla

#### Microtrichia sensilla

MT were hair-like, numerous and distributed on the scape, pedicel, and flagellum of the antennae. Some were also present on the rest of the whitefly body. These sensilla had a broad base and gradually tapered to the tip, which was bulbous. The tips of all MT pointed to the top of the antennae (Fig 2). MT on the scape and pedicel grew on smooth skin while those on the flagella were organized in a circle around the antennae. Some of these circum sensilla groups looked like dumbbells (Fig 6C).

#### Grooved surface trichodea sensilla

These hair-like structures were only present in the dorsal scape joints. Their base slightly protruded into the cuticle, without a base socket but with uniformly grooved surface. These grooves extended to the anterior, parallel to the axis of the sensilla (Fig 5B). Only one GT, was found in the antennae of both sexes in each species and its length was around 7.88 μm.

#### Chaetae sensilla

These smooth sensilla were present on the pedicel of both sexes in the two species, and were concentrated on the ventral surface. Each chaetae sensilla had a circle socket base that was 1.66 μm high, 2.89 μm wide, and had an inner diameter of 0.88 μm and an outer diameter of 1.50 μm. Their length was about 9.58 μm (except base), which is longer than the MT (Fig 5C and 5D). In the females of both MEAM 1 and MED species, 5 CH were found and in the males 7 were present. In the males, 1 in 7 CH was present on the dorsal surface of the pedicel, the remaining 6 sensilla were found on the other ventral of the of bulbous pedicel. These sensilla were also present on the other areas of the whitefly body, particularly the head, but not on the scape.

#### Coeloconic sensilla

These daisy-like sensilla were composed of a central peg set on the floor of a relatively shallow depression, and were surrounded by inwardly directed MT (Fig 6A and 6B). In total, 4 CO were present on the ventral surface towards the anterior end of the sub-segments (2 in the first flagellum, 1 in the third flagellum, and 1 in the fifth flagellum). No differences were observed in the number and distribution of these sensilla between the 2 cryptic species and sexes. CO in the first flagellum were bigger and shorter (CO I, about high 1.77 μm and width 1.63 μm, Fig 6C) than those in other locations (CO II, about high 2.43 μm and width 1.49 μm, Fig 6D).

#### Basiconic sensillaⅠ

Basiconic sensilla Ⅰ, Ⅱand III were cone-shaped pegs with a pitted appearance (Figs 2–4). They were observed on the first, fourth and fifth flagellum. They were always positioned anteriorly on the ventral surface of the sub-segment. There was no difference in the distribution and number of these sensilla among the 2 cryptic species and sexes.

Basiconic sensilla Ⅰwere robust on the antennae, grew in a big basal socket, which had several MT (Fig 2). BA Ⅰ in MEAM1 females and males had multiple-pitted surface, and were formed by clutter gullies, like linen. They were 10.12 μm in length in both sexes, and had 2.45 μm and 2.08 μm width in the females and males, respectively. BA Ⅰon the antennae of MED females and males were different from MEAM1, which had multiple pockings, and was 7.56 μm in length, and had 2.42 μm and 1.92 μm width in MED females and males respectively. Both were shorter than MEAM1. This sensillum only exists on the first flagellum.

#### Basiconic sensillaⅡ

The BA Ⅱ grew in twos in a single basal socket. One was longer than the other with the shorter one beneath the longer one. The shorter sensillum was whole covered by the longer one. The surface of the longer one had several pits in MEAM1, while the shorter one in both species and the longer one on MED had smooth surfaces. The basal socket was bell-shapped with a blunt tip (Fig 3). Lengths of the long and short sensilla were not different between MEAM1 and MED flies. The longer one was 5.18 μm long, and the shorter one was 1.56 μm. The single BA Ⅱ was present only on the ventral surface of the fourth flagellum; however, there were several MT on the outside of the basal socket.

#### Basiconic sensilla Ⅲ

These cement-like surface sensilla were the longest in the antennae and were present only in the fifth flagellum (Fig 4). These sensilla in *B*. *tabaci* MED extended from the cuticle, and were 11.11 μm long and had no base socket. They were also shorter than that in MEAM1, which was 13.24 μm long and had a base socket.

#### Finger-like sensilla

These sensilla were small pegs, finger-like and had smooth surface at the distal end of the fifth flagellum in both species and sexes studied (Figs 6C and 6D). The sensilla socket completely wrapped its base, which was formed directly by the antenna socket. These FS extended from the interior of the base. Sexual dimorphism was not observed in the FS in the *B*. *tabaci* cryptic species, MEAM1 and MED. These sensilla in MEAM 1 were 8.59 μm long and 1.53 μm wide, and were bigger than that in MED, which were 6.19 μm long and 1.38 μm wide.

## Discussion

Our observations on the number of antennal sensilla in the cryptic *B*. *tabaci* species, MEAM1 and MED, revealed that no differences in the number and distribution of antenna sensilla in MEAM 1 and MED antennae. However, surface structure of the BA I differed between the two species with MEAM 1 showing a multiple-pitted linen surface and MED having a multiple pocking surface. Interestingly, we observed for the first time the presence of GT and BA II on *B*. *tabaci* flies.

### Microtrichia sensilla

The MT was the most widely distribubed sensilla on the surface of *B*. *tabaci*, and was spread all over the antennae and the whole body. Previously, Hill [17] and Gupta [18] described the MT as hairs and setea, which are distributed on the peidcel of *B*. *tabaci* and *T*. *Vaporariorum*, and the fifth flagellar sub-segment in *B*. *tabaci*. Our observations are consistent with the comprehensive description of these sensilla by Mellor and Anderson [14]. These hair-like stuctures may play a role in holding or hooking the insects in place, while the bulbous hairs may help the insects stay away from the leaf that prevents their trapping into leaves with honeydew.

### Grooved surface Trichodea

GT are the most widely distribubed sensilla that are also present in large numbers on some insects such as *Ostrinia nubilalis* [19, 20]. However, there are no previous reports about this type of sensilla on *B*. *tabaci*, and this is the first report of the existence of one GT at the base of the scape in *B*. *tabaci* MEAM1 and MED. Zhou et al [21] studied this sensilla in *Encarsia guadeloupae* antenna and divided them into two classes, one was non-porous sensilla trichodea (ST-NP) and the other had longitudinal grooves. Our observations are consistent with this description. Onagbola [22] divided the sensilla in the *Pteromalus cerealellae* antenna into four classes. Among them, sensilla trichodea type II is similar to our observations in *B*. *tabaci*. Similarly, the sensilla trichodea of *Encarsia sophia* [23] are also consistent with this study. Previous studies showed that GT functioned as mechanical receptors that can feel the direction of the wind flow through touch or vibration [24]. GT only exist on the dorsal medial side in *B*. *tabaci* scape, which is close to the junction between the antenna and head. Since the tip of this sensillum could reach the head, we presume that the mechanical function may play a role in regulating the direction of *B*. *tabaci* movement towards that host plant.

### Chaetae sensilla

This type of sensilla was described as very stiff with upward convex coxacava [14, 17]. We found that CH exist on the bulbous pedicel of both cryptic *B*. *tabaci* species. Previous studies [17, 18] mentioned its presence in *B*. *tabaci* on the ventral surface of the pedicel, but did not give a detailed description. Aljunid and Anderson [25] reported that chaetae sensilla in *Nilaparvata lugens* antennae are more common on the ventral surface. However, the location of the CH in our study is consistent with Mellor and Anderson [14], who reported their presence in bulbous pedicel. However, the number of CH in male *B*. *tabaci* MEAM1 and MED we observed are different from their study; they showed only 5 chaetae sensilla in *B*. *tabaci* and did not mention the differences between males and females. Our results showed that there are 5 CH in female and 7 in male MEAM1 and MED cryptic species although no structural differences were observed between the two species. Chaetae sensilla could perceive the movement of antennae as proprio receptors as shown previously [21, 26]. Frazier [27] described that most chaetae are presumed to have tactile function. Based on our observation that the numbers of these sensilla in males were more than in females, we presume that it could be involved in finding a mate by the males.

### Coeloconic sensillaⅠandⅡ

CO start in a round cave and like a peg with longitudinal grooves. Ciliate arise in round edges and have a protective effect on CO. In *B*. *tabaci* MEAM1 and MED, these sensilla are analogous to other whiteflies and insects [14, 28]. Previously, these sensilla were described as rhinaria by Domenichini [13] and lachneat by Bink [29] and sensilla by others [30]. We divided CO into two classes, CO Ⅰand Ⅱ. CO Ⅰ was stubby and was distributed on the first and fifth flagella. There were 3 CO Ⅰ on each antenna of *B*. *tabaci* MEAM1 and MED. CO Ⅱ was longer and thinner than CO Ⅰ, and only one was present on the third flagellum. This is also a first report. The CO in *Drosophila* antennae have highly specialized neurons and perform chemosensory function [31], while in other insects it is reported to have olfactory function (Van Baaren et al, 1996) and chemo- and thermo-receptor functions [26,31–37].

### Basiconic sensillaⅠ, Ⅱ, and Ⅲ

Previous studies have shown that BA are large sensilla with papula surface, sensory cones, and extensively pitted surface [13,14,29,30]. Our SEM images showed that there are three BA on *B*. *tabaci* MEAM1 and MED, each with different surface structure and/or numbers. This is the first report of three types of BA in *B*. *tabaci*, and varies from previous studies [13,14,29,30]. We also found that these sensilla are different between *B*. *tabaci* MEAM1 and MED. BAⅠwas located at the end of the first flagellum. In MEAM1, it had a linen-like surface, while in MED it had a multiple pocking-like surface. BAⅡ is unique, and has not been reported in other insects. This was a double BA with one long and one short, located in the same socket of the fourth flagellum. Since the short one was under the long one, it is presumed that the long one may protect the shorter one. The long one had a pitted surface in MEAM1, but the short and long ones in MED were relatively smooth. BA Ⅲ is the longest sensilla in *B*. *tabaci* antennae with their length in MEAM1 longer than in MED. Their surfaces were not smooth but rough like concrete. The rough surface could be to expand the surface area to function as an effective mechanical receptor and for olfaction [38–40]. It was previously reported to influence the courtship behavior by detecting pheromones [40]. Therefore, we presume that the BAⅡ in the two cryptic *B*. *tabaci* species could enhance courtship behavior and olfaction.

### Finger-like sensilla

This sensillum was described as terminal hair chaetae sensilla comparable to the chaetae on the pedicel in previous studies [14]. Based on our observations, we suggest that it has a finger-like from in its external morphology. Further, its location suggests a mechano-sensitive receptor function [33] that enables reception of stimuli from the wing flaps of the host.

The types and numbers of sensilla were similar between the two cryptic *B*. *tabaci* species, MEAM1 and MED (Table 2). However, their structures were different. Through SEM, we discovered new sensilla, and distinguished the difference between *B*. *tabaci* MEAM1 and MED. The previous reference showed the different sensilla on the antennae of *B*. *tabaci* cryptic species play different roles in the behavior of this insect pest and may primarily function as mechanoreceptors and/or chemoreceptors [39], the functions should be explored more in future. But our results could further the study of olfactory mechanisms in insects and provide the basis to link morphology to the behavior and taxonomy of insects. This will also help regulate the behavior of *B*. *tabaci* and to devise novel methods for integrated pest management.
